# NoMAS: A Computational Approach to Find Mutated Subnetworks Associated With Survival in Genome-Wide Cancer Studies

**DOI:** 10.3389/fgene.2019.00265

**Published:** 2019-04-10

**Authors:** Federico Altieri, Tommy V. Hansen, Fabio Vandin

**Affiliations:** ^1^Department of Information Engineering, University of Padova, Padova, Italy; ^2^Department of Mathematics and Computer Science, University of Southern Denmark, Odense, Denmark

**Keywords:** cancer genomics, survival analysis, network analysis, log-rank statistic, holdout approach

## Abstract

Next-generation sequencing technologies allow to measure somatic mutations in a large number of patients from the same cancer type: one of the main goals in their analysis is the identification of mutations associated with clinical parameters. The identification of such relationships is hindered by extensive genetic heterogeneity in tumors, with different genes mutated in different patients, due, in part, to the fact that genes and mutations act in the context of *pathways*: it is therefore crucial to study mutations in the context of interactions among genes. In this work we study the problem of identifying subnetworks of a large gene-gene interaction network with mutations associated with survival time. We formally define the associated computational problem by using a score for subnetworks based on the log-rank statistical test to compare the survival of two given populations. We propose a novel approach, based on a new algorithm, called Network of Mutations Associated with Survival (NoMAS) to find subnetworks of a large interaction network whose mutations are associated with survival time. NoMAS is based on the color-coding technique, that has been previously employed in other applications to find the highest scoring subnetwork with high probability when the subnetwork score is additive. In our case the score is not additive, so our algorithm cannot identify the optimal solution with the same guarantees associated to additive scores. Nonetheless, we prove that, under a reasonable model for mutations in cancer, NoMAS identifies the optimal solution with high probability. We also design a holdout approach to identify subnetworks significantly associated with survival time. We test NoMAS on simulated and cancer data, comparing it to approaches based on single gene tests and to various greedy approaches. We show that our method does indeed find the optimal solution and performs better than the other approaches. Moreover, on three cancer datasets our method identifies subnetworks with significant association to survival when none of the genes has significant association with survival when considered in isolation.

## 1. Introduction

Recent advances in next-generation sequencing technologies have enabled the collection of sequence information from many genomes and exomes, with many large human and cancer genetic studies measuring mutations in all genes for a large number of patients of a specific disease (Cancer Genome Atlas Research Network, 2013, 2014; Cancer Genome Atlas Network, 2015; Cancer Genome Atlas Research Network et al., 2017; Raphael et al., 2017). One of the main challenges in these studies is the interpretation of such mutations, in particular the identification of mutations that are clinically relevant. For example, in large cancer studies one is interested in finding somatic mutations that are associated with survival and that can be used for prognosis and therapeutic decisions. One of the main obstacles in finding mutations that are clinically relevant is the large number of mutations present in each cancer genome. Recent studies have shown that each cancer genome harbors hundreds or thousands of somatic mutations (Garraway and Lander, 2013), with only a small number (e.g., ≤ 10) of *driver* mutations related to the disease, while the vast majority of mutations are *passenger*, random mutations that are accumulated during the process that leads to cancer but not related to the disease (Vogelstein et al., 2013).

In recent years, several computational and statistical methods have been designed to identify driver mutations and distinguish them from passenger mutations, exploiting data from large cancer studies (Raphael et al., 2014). Many of these methods analyze each gene in isolation, and use different single gene scores (e.g., mutation frequency, clustering of mutations, etc.) to identify significant genes (Dees et al., 2012; Lawrence et al., 2013; Tamborero et al., 2013). While useful in finding driver genes, these methods suffer from the extensive *heterogeneity* of mutations in cancer, with different patients showing mutations in different cancer genes (Kandoth et al., 2013). One of the reasons of such mutational heterogeneity is the fact that driver mutations do not target single genes but rather *pathways* (Vogelstein et al., 2013), groups of interacting genes that perform different functions in the cell. Several methods have been recently proposed to identify significant groups of interacting genes in cancer (Vandin et al., 2012b; Hofree et al., 2013; Kim et al., 2015; Leiserson et al., 2015a,b; Shrestha et al., 2017). Many of these methods integrate mutations with interactions from genome-scale interaction networks, without restricting to already known pathways, that would hinder the ability to discover new important groups of genes.

In addition to mutation data, large cancer studies often collect also clinical data, including survival information, regarding the patients. An important feature of survival data is that it often contains *censored* measurements (Kalbfleisch and Prentice, 2002): in many studies a patient may be alive at the end of the study or may leave the study before it ends, therefore only a lower bound to the survival of the patient is known. Survival information is crucial in identifying mutations that have a clinical impact. However, the survival information is commonly used only *after* candidate genes or groups of genes have been identified using other methods, as the ones described above, to evaluate the clinical significance of such genes or groups of genes (Cancer Genome Atlas Research Network, 2011; Hofree et al., 2013). Overall, there is a lack of methods that integrate mutations, interaction information, and survival data to directly identify groups of interacting genes associated with survival.

The field of survival analysis has produced an extensive literature on the analysis of survival data, in particular for the comparison of the survival of two given populations (sets of samples) (Kalbfleisch and Prentice, 2002). The most commonly used test for this purpose is the log-rank test (Mantel, 1966; Peto and Peto, 1972). In genomic studies we are not given two populations, but a single set of samples, and are required to identify mutations that are associated with survival. The log-rank test can be used to this end to identify single genes associated with survival time by comparing the survival of the patients with a mutation in the gene with the survival of the patients with no mutation in the gene. The other commonly used test, the Cox Proportional-Hazards model (Kalbfleisch and Prentice, 2002), is equivalent to the log-rank test when the association of a binary feature with survival is tested, as it is in the case of interest to genomic studies. For a given group of genes, one can *assess* the association of mutations in the genes of the group with survival by comparing the survival of the patients having a mutation in at least one of the genes with the survival of the patients with no mutation in the genes. However, this approach cannot be used to *discover* sets of genes, since one would have to screen all possible subsets of genes and test their association with survival, and the number of subsets of genes to screen is enormous even considering only groups of genes interacting in a protein interaction network (e.g., there are >10^15^ groups of 8 interacting genes in HINT+HI2012 network; Leiserson et al., 2015b).

In this paper we study the problem of finding sets of interacting genes with mutations associated to survival using data from large cancer sequencing studies and interaction information from a genome-scale interaction network. We focus on the widely used log-rank statistic as a measure of the association between mutations in a group of genes and survival. Our contribution is in five parts: first, we formally define the problem of finding the set of *k* genes whose mutations show the maximum association to survival time by using the log-rank statistic as a score for a set of genes: we show that such problem is NP-hard. We show that the problem remains hard when the set of *k* genes is required to form a connected subnetwork in a large graph with at least one node of large degree (*hub*). Second, we propose an efficient algorithm, Network of Mutations Associated with Survival (NoMAS), based on the color-coding technique, to identify subnetworks associated with survival time. Color-coding has been previously used to find high scoring graphs for bioinformatics applications (Dao et al., 2011; Hormozdiari et al., 2015) when the score for a subnetwork is *set additive* (i.e., the score of a subnetwork is the sum of the scores of the genes in the subnetwork). In our case the log-rank statistic is not set additive, and we prove that there is a family of instances for which our algorithm cannot identify the optimal solution. Nonetheless, we prove that, under a reasonable model for mutations in cancer, our algorithm identifies the optimal solution with high probability. Third, we test our algorithm on simulated data and on data from three large cancer studies from The Cancer Genome Atlas (TCGA). On simulated data, we show that our algorithm does find the optimal solution while being much more efficient than the exhaustive algorithm that screens all sets of genes. On cancer data, we show that our algorithm finds the optimal solution for all values of *k* for which the use of the exhaustive algorithm is feasible, and identifies better solutions (in terms of association to survival) than a greedy algorithm similar to the one used in Reimand and Bader (2013). Fourth, to strengthen the statistical reliability of NoMAS's results, we employ a holdout scheme, splitting the patients dataset in two parts, a *training* set and a *holdout* set. While solutions of the NoMAS are computed on the former, the assessments of their statistical significance are performed on the latter, thus providing a correction for the multiple hypothesis testing performed on the training set. Finally, we show that NoMAS identifies better solutions than using an (additive) score (i.e., the same gene score used in Vandin et al., 2012a) for a set of genes. For the cancer datasets, we show that our algorithm identifies novel groups of genes associated with survival where none of them is associated with survival when considered in isolation. The work is organized as follows: in section 2 we provide the description of the model and NoMAS; section 3 presents the analysis of the algorithm (section 3.1), including the analysis under a reasonable model for mutations in cancer and analysis of our experiments on both simulated and real data (section 3.2); finally section 4 presents the discussion of our results. Details for our theoretical results are given in Supplementary Material.

## 2. Materials and Methods

In this section we present the model we consider, our algorithm NoMAS, and the tests we have designed to assess the statistical significance of the results.

### 2.1. Computational Problem

In survival analysis, we are given two populations (i.e., sets of samples) *P*_0_ and *P*_1_, and for each sample *i* ∈ *P*_0_ ∪ *P*_1_ we have its survival data: i) the survival time *t*_*i*_ and ii) the censoring information *c*_*i*_, where *c*_*i*_ = 1 if *t*_*i*_ is the exact survival time for sample *i* (i.e., sample *i* is not censored), and *c*_*i*_ = 0 if *t*_*i*_ is a lower bound to the survival time for sample *i* (i.e., sample *i* is censored). Let *m*_0_ be the number of samples in *P*_0_, *m*_1_ be the number of samples in *P*_1_, and *m* = *m*_0_ + *m*_1_ be the total number of samples. Without loss of generality, the samples are {1, 2, …, *m*}, the survival times are *t* = 1, 2, …, *m*, with *t*_*i*_ = *i* (i.e., the samples are sorted by increasing values of survival), and we assume that there are no ties in survival times. The survival data is represented by two vectors **c** and **x**, with *c*_*i*_ representing the censoring information for sample *i*, and *x*_*i*_ represents the population information: *x*_*i*_ = 1 if sample *i* is in population *P*_1_, and *x*_*i*_ = 0 otherwise. Given the survival data for two populations *P*_0_ and *P*_1_, the significance in the difference of survival between *P*_0_ and *P*_1_ can be assessed by the widely used log-rank test (Mantel, 1966; Peto and Peto, 1972). The log-rank statistic is

(1) $$\begin{array}{l} V\left(\text{x},\text{c}\right)=\overset{m}{\underset{j=1}{\sum }}c_{j}\left(x_{j}-\frac{m_{1}-\sum_{i=1}^{j-1}x_{i}}{m-j+1}\right) \end{array}$$

Under the (null) hypothesis of no difference in survival between *P*_0_ and *P*_1_, the log-rank statistic asymptotically follows a normal distribution $N\left(0,\sigma^{2}\right)$, where the standard deviation^1^ is given by: $\sigma \left(\text{x},\text{c}\right)=\sqrt{\frac{m_{0}m_{1}}{m\left(m-1\right)}\left(\left(\overset{m}{\underset{j=1}{\sum }}c_{j}\right)-\overset{m}{\underset{j=1}{\sum }}c_{i}\frac{1}{m-j+1}\right)}.$ Thus the normalized log-rank statistic, defined as $\frac{V\left(\text{x},\text{c}\right)}{\sigma \left(\text{x},\text{c}\right)}$, asymptotically follows a standard normal N(0,1) distribution, and the deviation of $\frac{V\left(\text{x},\text{c}\right)}{\sigma \left(\text{x},\text{c}\right)}$ from 0 is a measure of the difference in survival between *P*_0_ and *P*_1_.

In genomic studies, we are given mutation data for a set G of *n* genes in a set P of *m* samples, represented by a mutation matrix *M* with *M*_*i, j*_ = 1 if gene *i* is mutated in patient *j* and *M*_*i, j*_ = 0 otherwise. We are also given survival data (survival time and censoring information) for all the *m* samples. Given a set S⊂G of genes, we can assess the association of mutations in the set S with survival by comparing the survival of the population $P_{1}^{S}$ of samples with a mutation in at least one gene of S and the survival of the population $P_{0}^{S}$ of samples with no mutation in the genes of S. That is, $P_{0}^{S}=\left\{j\in P:\underset{i\in S}{\sum }M_{i,j}=0\right\}$ and $P_{1}^{S}=\left\{j\in P:\underset{i\in S}{\sum }M_{i,j}>0\right\}$.

Given the set G of all genes for which mutations have been measured, we are interested in finding the set S⊂G with |S|=k that has maximum association with survival by finding the set S that maximizes the absolute value of the normalized log-rank statistic. Given a set S of genes, let ${\text{x}}^{S}$ be a 0−1 vector, with $x_{i}^{S}=1$ if at least one gene of S is mutated in patient *i*, and $x_{i}^{S}=0$ otherwise. The normalized log-rank statistic for the set S is then $\frac{V\left({\text{x}}^{S},\text{c}\right)}{\sigma \left({\text{x}}^{S},\text{c}\right)}$. Note that for a given set of patients the censoring information **c** is fixed, therefore we can consider the log-rank statistic as a function $V\left({\text{x}}^{S}\right)$ of ${\text{x}}^{S}$ only. Analogously, we can rewrite $\sigma \left({\text{x}}^{S},\text{c}\right)=\sigma \left({\text{x}}^{S}\right)f\left(\text{c}\right)$, where $\sigma \left({\text{x}}^{S}\right)=\sqrt{m_{1}\left(m-m_{1}\right)}$ with $m_{1}=|P_{1}^{S}|$, and $f\left(\text{c}\right)=\sqrt{\frac{1}{m\left(m-1\right)}\left(\left(\overset{m}{\underset{j=1}{\sum }}c_{j}\right)-\overset{m}{\underset{j=1}{\sum }}c_{j}\frac{1}{m-j+1}\right)}$ does not depend on **x**^*S*^ and is fixed given **c**.

To identify the set of *k* genes most associated with survival, we can then consider the score $\left|w\left(S\right)|=|\frac{V\left({\text{x}}^{S}\right)}{\sigma \left({\text{x}}^{S}\right)}\right|$. For ease of exposition in what follows we consider the score w(S), corresponding to a one tail log-rank test for the identification of sets of genes with mutations associated with reduced survival; the identification of sets of genes with mutations associated with increased survival is done in an analogous way by maximizing the score -w(S). We define the following problem.

**The max** *k***-set log-rank problem:** *G*iven a set G of genes, an *n* × *m* mutation matrix *M* and the survival information (time and censoring) for the *m* patients in *M*, find the set S⊂G of *k* genes maximizing w(S).

We have the following.

**Theorem 1**. *The max *k*-set log-rank problem is NP-hard*.

We now define the max connected *k*-set log-rank problem that is analogous to the max *k*-set log-rank problem but requires feasible solutions to be connected subnetworks of a given graph *I*, representing gene-gene interactions.

**The max connected** *k***-set log-rank problem:** *G*iven a set G of genes, a graph I=(G,E) with E⊆G×G, an *n* × *m* mutation matrix *M* and the survival information (time and censoring) for the *m* patients in *M*, find the set S of *k* genes maximizing w(S) with the constraint that the subnetwork induced by S in *I* is connected.

If *I* is the complete graph, the max connected *k*-set log-rank problem is the same as the max *k*-set log-rank problem. Thus, the max connected *k*-set log-rank problem is NP-hard for a general graph. However, we can prove that the problem is NP-hard for a much more general class of graphs.

**Theorem 2**. *The max connected k-set log-rank problem on graphs with at least one node of degree* $O\left(n^{\frac{1}{c}}\right)$, *where c* > 1 *is constant, is NP-hard*.

### 2.2. Algorithm NoMAS

We design a new algorithm, Network of Mutations Associated with Survival (NoMAS)^2^, to solve the max connected *k*-set log-rank problem. The algorithm is based on an adaptation of the color-coding technique (Alon et al., 1994). Our algorithm is analogous to other color-coding based algorithms that have been used before to identify subnetworks associated with phenotypes in other applications where the score is additive (Dao et al., 2011; Hormozdiari et al., 2015).

**Figure 1:**
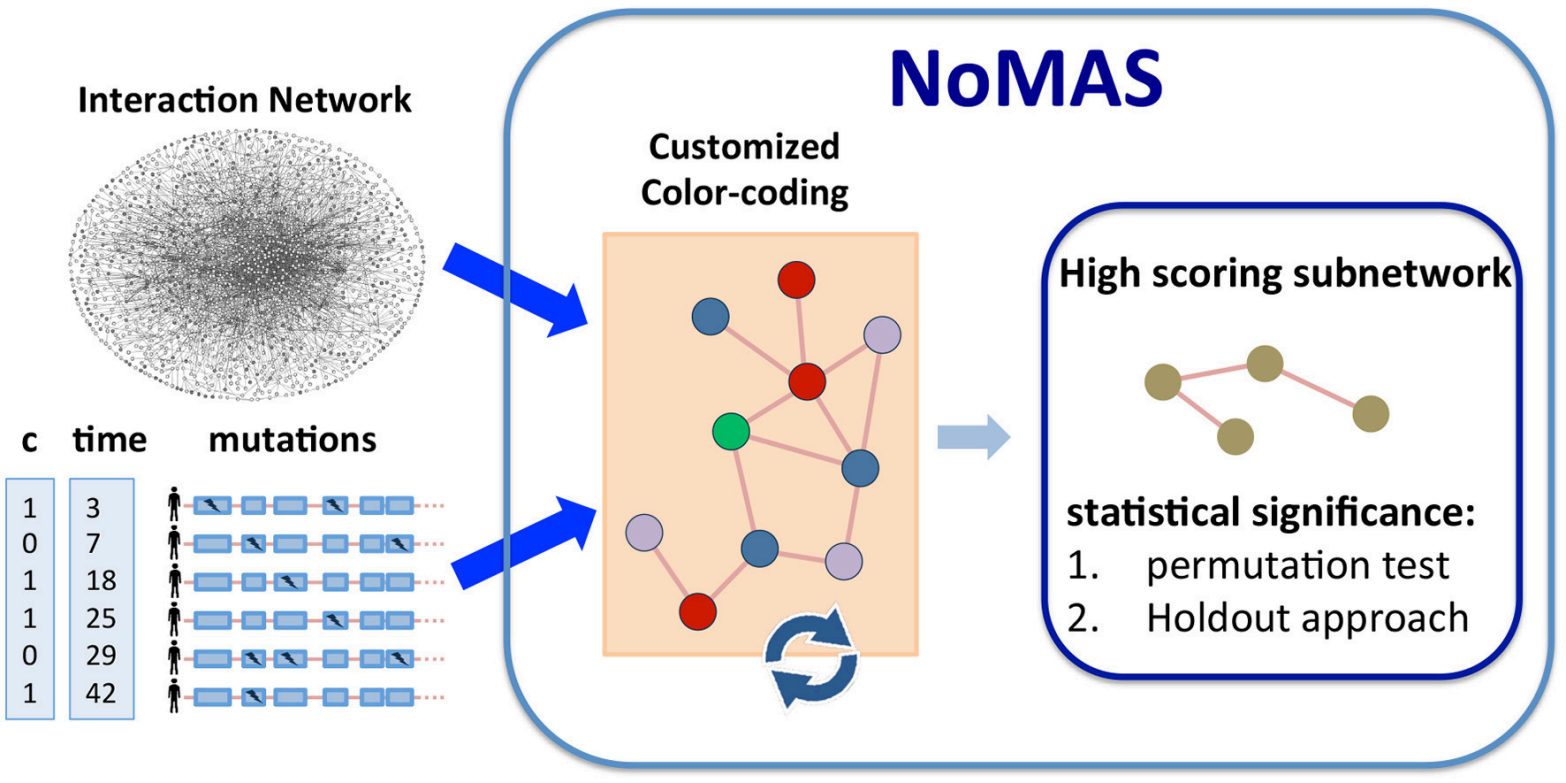
Algorithm NoMAS. Given alteration data and survival information (time and censoring status) for a set of patients, NoMAS employs a color coding approach to identify subnetworks with mutations associated with survival time, i.e., with high log-rank statistic, and then assesses the statistical significance of the subnetworks using (i) permutation testing and (ii) a holdout approach.

Figure 1 provides an overview of NoMAS. The input to NoMAS is an undirected graph *G* = (*V, E*), an *n* × *m* mutation matrix *M*, and the survival information **x**, **c** for the *m* patients in *M*. NoMAS first identifies a subnetwork S with high weight $\frac{w\left({\text{x}}^{S}\right)}{\sigma \left({\text{x}}^{S}\right)}$. To identify a subnetwork of high weight, the algorithm proceeds in iterations. In each iteration NoMAS colors *G* with *k* colors by assigning to each vertex *v* a color C(v)∈{1,…,k} chosen uniformly at random. For a given coloring of *G*, a subnetwork S is said to be *colorful* if all vertices in S have distinct colors. The *colorset* of S is the set of colors of the vertices in S. Note that the number of different colorsets (subsets of {1, …, *k*}) is 2^*k*^. In each iteration the algorithm efficiently identifies high-scoring colorful subnetworks, and at the end the highest-scoring subnetwork among all iterations is reported.

Consider a given coloring of *G*. Let *W* be a (2^*k*^ − 1) × |*V*| table with a row for each non-empty colorset and a column for each vertex in *G*. Entry *W*(*T, u*) stores the set of vertices of one connected colorful subnetwork that has colorset *T* and includes vertex *u*. Entries of *W* can be filled by dynamic programming. For colorsets of size 1, the corresponding rows in *W* are filled out trivially: *W*({α}, *u*) = {*u*} if α=C(u), and *W*({α}, *u*) = ∅ otherwise.

For entry *W*(*T, u*) with |*T*| ≥ 2, NoMAS computes *W*(*T, u*) by combining a previously computed *W*(*Q, u*) for *u* with another previously computed *W*(*R, v*) where *v* is a neighbor of *u* in *G*, ensuring that the resulting subnetwork is connected and contains *u*. Colorfulness is ensured by selecting *Q* and *R* such that *Q* ∩ *R* = ∅ and *Q* ∪ *R* = *T*, and in turn ensures that *W*(*T, u*) contains |*T*| distinct vertices. Note that for a given *T* the choice of *Q* uniquely defines *R*. Thus, for each neighbor *v* of *u* there are (at most) 2^|*T*|−1^ possible combinations. Let $S^{\prime }\left(T,u\right)$ be the set of all colorful subnetworks that can be obtained by combining an entry *W*(*Q, u*) for *u* and an appropriate entry *W*(*R, v*) for a neighbor *v* of *u* so that *Q* ∪ *R* = *T, Q* ∩ *R* = ∅. That is: $S^{\prime }\left(T,u\right)={⋃}_{\begin{array}{c} v:\left(u,v\right)\in E\\ Q\cup R=T,Q\cap T=\emptyset \end{array}}\left\{W\left(Q,u\right)\cup W\left(R,v\right)\right\}$(in the definition of $S^{\prime }\left(T,u\right)$ we assume that the union with ∅ returns ∅). *W*(*T, u*) stores the element of $S^{\prime }\left(T,u\right)$ with largest value of our objective function, that is $W\left(T,u\right)={\mathrm{argmax}}_{S\in S^{\prime }\left(T,u\right)}w\left(S\right).$ At the end, the best solution is identified by finding the entry of *W* of maximum weight. Analogously, NoMAS identifies sets that minimize w(S) (sets associated to increased survival) by maximizing the score -w(S). (See Appendix for pseudo code and illustrations of the working of NoMAS).

#### Parallelization

The computation of *W* is parallelized using *N* ≤ |*V*| processors. All entries of *W* are kept in shared memory and |*V*|/*N* unique columns uniformly at random are assigned to each processor. Entries of *W* are computed in order of increasing colorset sizes. We define the *i*-th *colorset group* as the set of all $\left(\begin{array}{c} k\\ i \end{array}\right)$ colorsets of size *i*. We exploit the fact that the rows within the *i*-th colorset group are computed by reading entries exclusively from rows belonging to colorset groups < *i*. When a processor has finished the rows of the *i*-th colorset group it waits for the other processors to do the same. When the last processor completes the *i*-th colorset group, all *N* processors can safely begin to compute rows of colorset group *i*+1. In total, *k* synchronization steps are carried out, one for each colorset group.

### 2.3. Statistical Significance

We designed two procedures to assess the statistical significance of the results found by NoMAS: the first is based on permutation testing, while the second uses a holdout approach.

#### Permutation Testing

After identifying the best solution S for the mutation matrix *M*, NoMAS can assess its statistical significance by i) estimating the *p*-value p(S) for the log-rank statistic (using a Monte-Carlo estimate with 10^8^ samples), and then ii) using a permutation test in which S is compared to the best solution $S^{p}$ for the mutation matrix *M*^*p*^ obtained by randomly permuting the rows of *M*. A total of 100 permutations are performed and the *permutation* p-value is recorded as the ratio of permutations in which $w\left(S^{p}\right)\geq w\left(S\right)$. While the *p*-value from the log-rank test reflects the association between mutations in the subnetwork and survival, the permutation *p*-value assesses whether a subnetwork with association with survival at least as extreme as the one observed in the input data can be observed when the genes are placed randomly in the network. Note that we can identify multiple solutions by considering different entries of *W* (even if the same solution may appear in multiple entries of *W*), and we obtain a permutation *p*-value for the *i*-th top scoring solution by comparing its score with the score of the *i*-th top scoring solution in the permuted datasets.

#### Holdout Method

We designed a holdout method to strengthen the statistical robustness of the results produced by NoMAS. We split the dataset in two parts, called *training* and *holdout*, and then run NoMAS on the former, obtaining subnetworks with high weight. The *p*-value of these subnetworks is then computed with a Monte-Carlo procedure estimate with 10^8^ samples on the holdout dataset. More in detail, assuming that a set *P* of *m* patients is analyzed, let *v* be a parameter with value in (0, 1) that represents the proportion of data in the training set: we partition *P* into two parts, *P*_*t*_ and *P*_*h*_, sized *m*_*t*_ = ⌊*mv*⌋ and *m*_*h*_ = *m* − *m*_*t*_ respectively. In order to preserve the survival distribution in both the training and the holdout set, the partition is performed over each of *g* temporal intervals of the same length, where *g* is a parameter to be passed in input by the user. The sets *P*_*t*_ and *P*_*h*_ are obtained by the union of the corresponding sets in each interval. Once we obtain the partition of *P* into *P*_*t*_ and *P*_*h*_, NoMAS is executed over the population *P*_*t*_ and *p*-value of the found solution is computed over *P*_*h*_.

## 3. Results

### 3.1. Analysis of NoMAS

We consider the performance of NoMAS excluding the statistical significance testing. The log-rank statistic w(S) is computed in time *O*(*m*_1_) ∈ *O*(*m*). The total time complexity for computing a single entry *W*(*T, u*) is then bounded by *O*(*m*deg(*u*)2^|*T*|−1^) ∈ *O*(*m*deg(*u*)2^*k*^), where *deg*(*u*) is the degree of *u* in *G*. Given a coloring of *G*, the computation of the entire table can thus be performed in time $O\left(2^{k}\underset{u\in V}{\sum }m\text{deg}\left(u\right)2^{k}\right)\in O\left(m|E|4^{k}\right)$. If *L* iterations are performed, then the complexity of the algorithm is *O*(*Lm*|*E*|4^*k*^).

Let *OPT* be the optimal solution. If the score w(S) was set additive, as the scores considered in previous applications of color-coding for optimization problems on graphs, to discover *OPT* it would be sufficient that *OPT* be colorful, that happens with probability *k*!/*k*^*k*^ ≥ *e*^−*k*^ for each random coloring. Therefore *O*(ln(1/δ)*e*^*k*^) iterations would be enough to ensure that the probability of *OPT* not being discovered is ≤ δ, resulting in an overall time complexity of *O*(*m*ln(1/δ)|*E*|(4*e*)^*k*^).

However, our score w(S) is not set additive [e.g., if two genes in S have a mutation in the same patient the weight of the patient is considered only once in w(S)]. Therefore, while *OPT* being colorful is still a necessary condition for the algorithm to identify *OPT*, the colorfulness of *OPT* is not a sufficient condition. In fact, we have the following.

**Proposition 1**. *For every *k* ≥ 3 there is a family of instances of the max connected *k*-set log-rank problem and colorings for which *OPT* is not found by NoMAS when it is colorful*.

Even more, we prove that when mutations are placed arbitrarily then for every subnetwork S and a given coloring of S, *any* color-coding algorithm that adds subnetworks of size *k* to *W* by merging neighboring subnetworks of size < *k* could be “fooled” to not add S to *W* by simply adding 3 vertices to *G* and assigning them a specific color.

**Theorem 3**. *For any optimal colorful connected subnetwork* S *of size k* ≥ 3 *and any color-coding algorithm* A *which obtains subnetworks with colorsets of cardinality i by combining 2 subnetworks with colorsets of cardinality < i, by adding 3 neighbors to* S *we have that* A *may not discover S*.

Intuitively, Proposition 1 and Theorem 3 show that if mutations are placed adversarially (and the optimal solution *OPT* has many neighbors), our algorithm may not identify *OPT*. However, we prove that our algorithm identifies the optimal solution under a generative model for mutations, that we deem the *Planted Subnetwork Model*. We consider w(S) as the unnormalized version of the log-rank statistic. In this model: i) there is a subnetwork D, |D|=k, with w(D)≥cm, for a constant *c* > 0; ii) each gene g∈D is such that $w\left(D\right)-w\left(D\backslash \left\{g\right\}\right)\geq \frac{c^{\prime }m}{k}$, for a constant *c*′& #x0003E; 0; iii) for each gene g∈D: *w*({*g*}) > 0; iv) for each gene ĝ∉D, ĝ is mutated with probability *p*_*g*_ in each patient, independently of all other events (and of survival time and censoring status).

Intuitively: (3.1) above states that the subnetwork D has mutations associated with survival; (3.1) states that each gene g∈D contributes to the association of mutations in D to survival; (3.1) states that each gene g∈D should have the same association to survival (increased or decreased) as D; and (3.1) states that all mutations outside D are independent of all other events (including survival time and censoring of patients).

We show that when enough samples are generated from the model above, our algorithm identifies the optimal solution with the same probability guarantee given by the color-coding technique for additive scores.

**Theorem 4**. *Let M be a mutation matrix corresponding to m samples from the Planted Subnetwork Model. If m* ∈ Ω(*k*^4^(*k* + ε)ln *n*) *for a given constant* ε > 0 *and O*(ln(1/δ)*e*^*k*^) *color-coding iterations are performed, then our algorithm identifies the optimal solution* D *to the max connected k-set log-rank with probability* $\geq 1-\frac{1}{n^{\varepsilon }}-\delta$.

### 3.2. Experimental Results

We assessed the performance of NoMAS by using simulated and cancer data. We compared NoMAS to the exhaustive algorithm that identifies the subnetwork of *k* vertices with the highest score *w*(*S*) for the values of *k* for which we could run the exhaustive algorithm (we implemented a parallelized version of the algorithm described in Maxwell et al., 2014 to efficiently enumerate all connected subnetworks), to three variants of a greedy algorithm similar to the one from Reimand and Bader (2013), and to the use of a score given by the sum of single gene scores. Cancer data is obtained from The Cancer Genome Atlas (TCGA). In particular, we consider somatic mutations (single nucleotide variants and small indels) for 268 samples of glioblastoma multiforme (GBM), 315 samples of ovarian adenocarcinoma (OV) and 174 samples of lung squamous cell carcinoma (LUSC) for which survival data is available.

For all our experiments we used as interaction graph *G* the graph derived from the application of a diffusion process on the HINT+HI2012 network^3^, a combination of the HINT network (Das and Yu, 2012) and the HI-2012 (Yu et al., 2011) set of protein-protein interactions, previously used in Leiserson et al. (2015a). The details of the diffusion process are described in Leiserson et al. (2015a). In brief, for two genes *g*_*i*_, *g*_*j*_ the diffusion process gives the amount of heat *h*(*g*_*i*_, *g*_*j*_) observed on *g*_*j*_ when *g*_*i*_ has one mutation, and the amount of heat *h*(*g*_*j*_, *g*_*i*_) observed on *g*_*i*_ when *g*_*j*_ has one mutation. The graph used for our analyses is obtained retaining an edge between *g*_*i*_ and *g*_*j*_ if max{*h*(*g*_*i*_, *g*_*j*_), *h*(*g*_*j*_, *g*_*i*_)} ≥ 0.012. The resulting graph has 9, 859 vertices and 42, 480 edges, with the maximum degree of a node being 438. In all our experiments we removed mutations in genes mutated in < 3 of the samples. For cancer data, this resulted in 890 mutated genes removed in GBM, 780 in OV, and 2, 915 in LUSC. The machine, on which all our experiments were carried out, consists of two CPUs of the type Intel Xeon E5-2698 v3 (2.30 GHz), each with 16 physical cores, for a total of 64 virtual cores, and 16 banks of 32 GB DDR4 (2,133 MHz) memory modules for a total of 512 GB of memory.

The remaining of the section is organized as follow: section 3.2.1 presents the results on simulated data, while section 3.2.2 presents the results on cancer data.

#### 3.2.1. Simulated Data

We assess the performance of NoMAS on simulated data generated under the Planted subnetwork Model. The subnetwork D⊂G,|D|=k associated with survival is generated by a random walk on the graph *G*. We model the association of D to survival by mutating with probability *p* one gene of D chosen uniformly at random in each sample among the $\frac{m}{4}$ of lowest survival. All other genes in D are mutated independently with probability 0.01 in all samples, to simulate passenger mutations (not associated with survival) in D (Lawrence et al., 2013). For genes in G\D, we used the same mutation frequencies observed in the GBM study, and mutate each gene independently of all other events.

**Figure 2:**
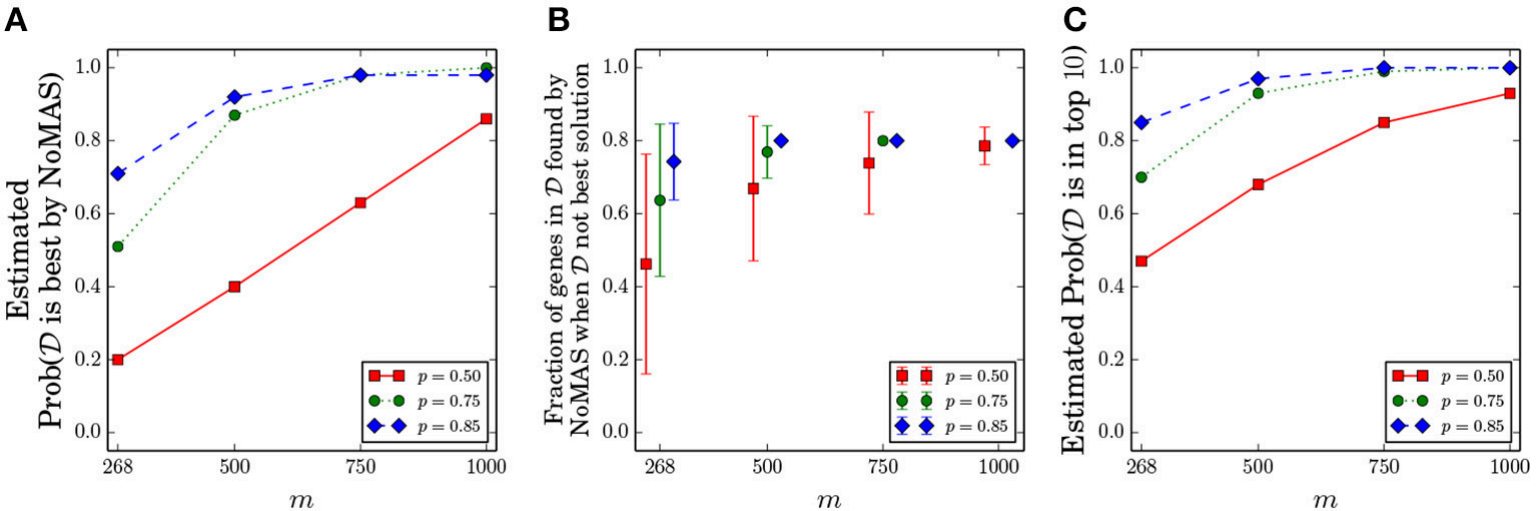
Results of NoMAS on simulated data from the Planted Subnetwork Model. One hundred datasets were generated for each pair (*m*,*p*), where *m* is the number of samples and for different probabilities *p* of mutations in the set D of genes associated with survival. **(A)** Probability that D is reported as the highest scoring solution by NoMAS. **(B)** Ratio of genes from the set D that are in the best solution when D is not the highest scoring solution by NoMAS. **(C)** Probability that D is among the top-10 solutions reported by NoMAS. All probabilities are estimated from the simulated datasets.

We fixed *k* = 5 and considered the values of *p* ∈ {0.5, 0.75, 0.85} and *m* ∈ {268, 500, 750, 1, 000}. We kept the same ratio of censored observations as in GBM and chose the censored samples uniformly among all samples. For every pair (*p, m*) we performed 100 simulations, running NoMAS on the dataset with *L* = 256 color-coding iterations, and recorded whether NoMAS reported D as the highest scoring subnetwork. Results are shown in Figure 2A. For sample sizes similar to the currently available ones, NoMAS frequently reports D as the highest scoring solutions when there is a quite strong association of D with survival (*p* ≥ 0.85), but for *m* = 1, 000 the highest scoring subnetwork reported by NoMAS is D in > 80% of the cases even for *p* = 0.5. Figure 2B shows that even when NoMAS does not report D as the highest scoring solution, the solution reported by NoMAS contains mostly genes that are in D, even for current sample size (e.g., on average 74% of the genes in the D are reported by NoMAS for *m* = 268 and *p* = 0.85 even when D is not the highest scoring solution by NoMAS). Finally, we assessed whether D would be among the highest scoring solutions in the table *W* computed by NoMAS: Figure 2C shows that by considering the top-10 solutions *W* the chances to identify D increase substantially even for *m* = 268 and *p* = 0.5, with most configurations having > 0.8 probability of finding D in the top-10 solutions by NoMAS. For a fixed *p* = 0.75 and for each value of *m* we assessed whether NoMAS identified the optimal solution even when it was not D (an event not excluded in the Planted subnetwork Model) and found that for *m* ≥ 500 NoMAS reported the optimal solution in 10 out of 10 cases (for *m* = 268 NoMAS identified the optimal solution 9 out of 10 times). These results show that NoMAS does indeed find the optimal solution in almost all cases even for sample sizes currently available (while the theoretical analysis of section 3.1 suggests that much larger sample sizes are required) and it can be used to identify D or the majority of it by considering the top-10 highest scoring solutions.

#### 3.2.2. Cancer Data

We assessed the performance of NoMAS on the GBM, OV, and LUSC datasets. We first assessed whether NoMAS identified the optimal solution by comparing the highest scoring solution reported by NoMAS with the one identified by using the exhaustive algorithm for *k* = 2, 3, 4, 5. In all cases we found that NoMAS does identify the optimal solution, while requiring much less running time compared to the exhaustive algorithm (Supplementary Figure 2). For *k* > 5 we could not run the exhaustive algorithm, while the runtime of NoMAS is still reasonable. The runtime of NoMAS can be greatly improved by using the parallelization strategy described in section 2.2 (Supplementary Figure 3). We therefore used NoMAS to find subnetworks of size *k* = 6 and *k* = 8. We also considered two modifications of NoMAS that solve some easy cases where NoMAS may not identify the highest scoring solution due to its subnetwork merging strategy (see Appendix for a description and pseudocode of the modifications). We run both modifications on GBM, OV, and LUSC for *k* = 6, 8 (using the same colorings used by the original version of NoMAS): in all cases the modified versions of NoMAS did not report subnetworks with higher scores than the ones from the original version of NoMAS. We also note that the original version of NoMAS is significantly faster in practice than its two modifications (Supplementary Figure 3) and, therefore, we used the original version of NoMAS in the remaining experiments.

**Figure 3:**
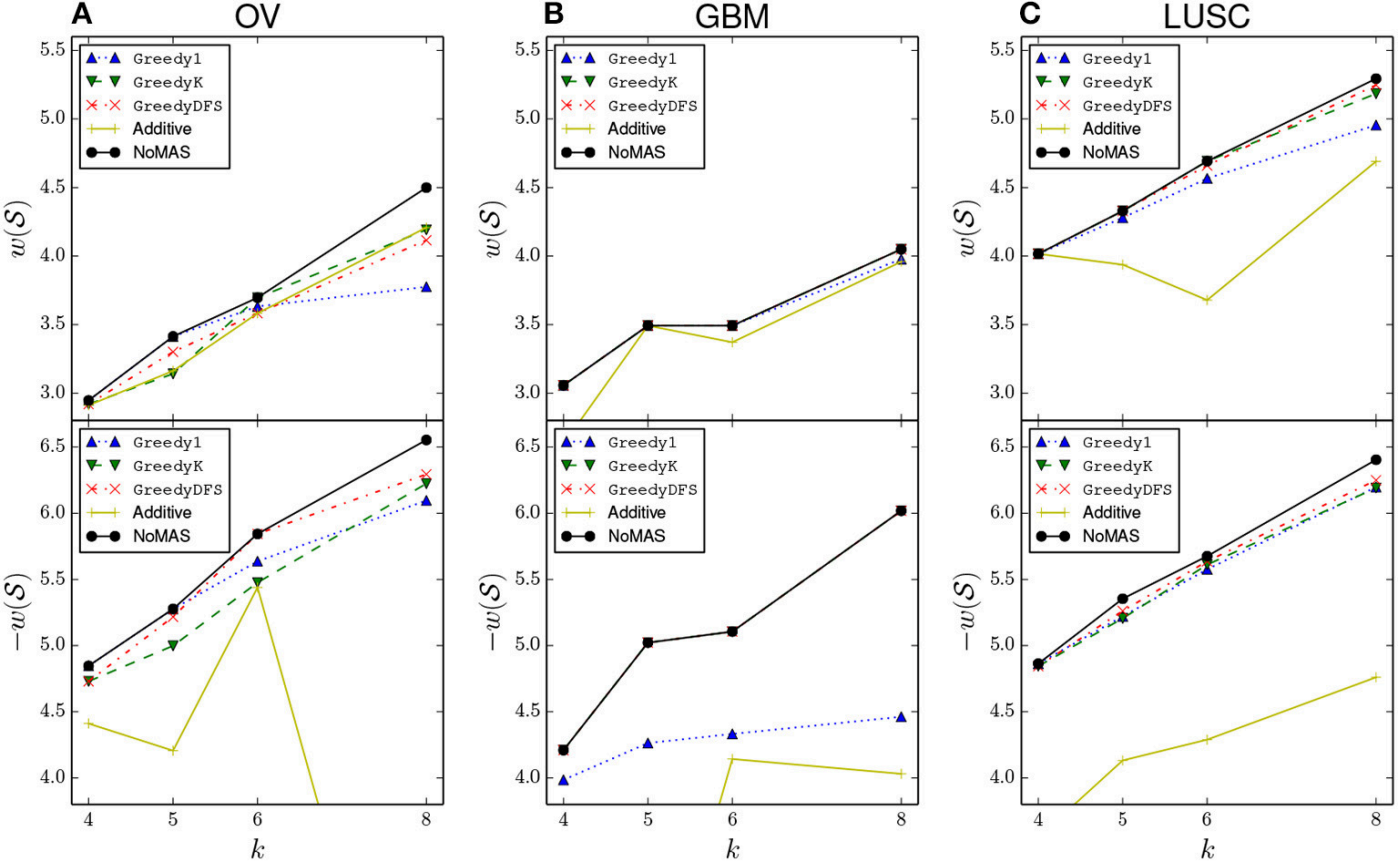
Comparison of the normalized log-rank statistic of the best solution reported by NoMAS, by greedy algorithms (see Appendix for the description), and by the algorithm that uses an additive scoring function a(S) (denote by “additive” in the plots). To maintain readability we omit values above −4.0 when considering mutations associated with increased survival. For each datasets the results for the maximization of w(S) (top panel) and the maximization of -w(S) (bottom panel) are shown separately. **(A)** Results for GBM dataset. **(B)** Results for OV dataset. **(C)** Results for LUSC dataset.

We also compared NoMAS with three different greedy strategies for the max connected *k*-set log-rank problem. All three algorithms build solutions starting from each node *u*∈*G* and, in iterations, by adding nodes to the current solution S, diversifying in the way they enlarge the current subnetwork S of size 1 ≤ *i* < *k*. (See Appendix for a description of the three greedy strategies). We run the three greedy algorithms on GBM, OV, and LUSC for *k* = 4, 5, 6, 8. For each dataset we compared the resulting subnetworks with the ones identified by NoMAS. Results are shown in Figure 3. In almost all cases we found that NoMAS discovered subnetworks with higher score than the subnetworks found by using greedy strategies, even if in some cases there is a greedy strategy that identifies the same subnetworks for all values of *k*. The difference in score increases as *k* increases, showing the ability of NoMAS to discover better solutions for larger values of *k*, with the main expense being the running time of NoMAS as opposed to the greedy strategies (Supplementary Figure 4). We also assessed whether the fact that greedy strategies discover lower scoring solutions than NoMAS has an impact on the estimate of the *p*-value in the permutational test. We considered the top-10 scoring solutions (corresponding to 10 different starting nodes *u* ∈ *G*) discovered by the best greedy strategy in the GBM dataset and computed the permutational *p*-value for each solution by generating 100 permuted datasets either using the (same) greedy strategy or NoMAS for (with only 32 iterations on the permuted data). Supplementary Figure 1 shows a comparison of the distribution of the *p*-values. As we can see, the greedy strategy incorrectly underestimate the permutational *p*-values for the solutions, due to the greedy algorithm not being able to identify solutions of score as high as NoMAS in the permuted datasets. The use of the greedy algorithms would then lead to both 1. identify solutions in real data with lower association to survival compared to NoMAS and 2. wrongly estimate their permutational *p*-value as more significant than it is.

Finally, we compared NoMAS with the use of an (additive) score that sums single gene scores (similar to the ones used in Vandin et al. (2012a). For each gene *g*∈*G* we computed the *p*-value *p*(*g*) for the association of *g* with survival using the log-rank test and defined $a\left(S\right)=\underset{g\in S}{\sum }-\log_{10}p\left(g\right)$. We then partitioned the genes according to their association with increased survival or with decreased survival and modified our algorithm to look for high scoring solutions in a partition using score a(S). Results are in Figure 3. We found that NoMAS outperforms the use of a single gene score, with a very large difference for certain values of parameters.

**Figure 4:**
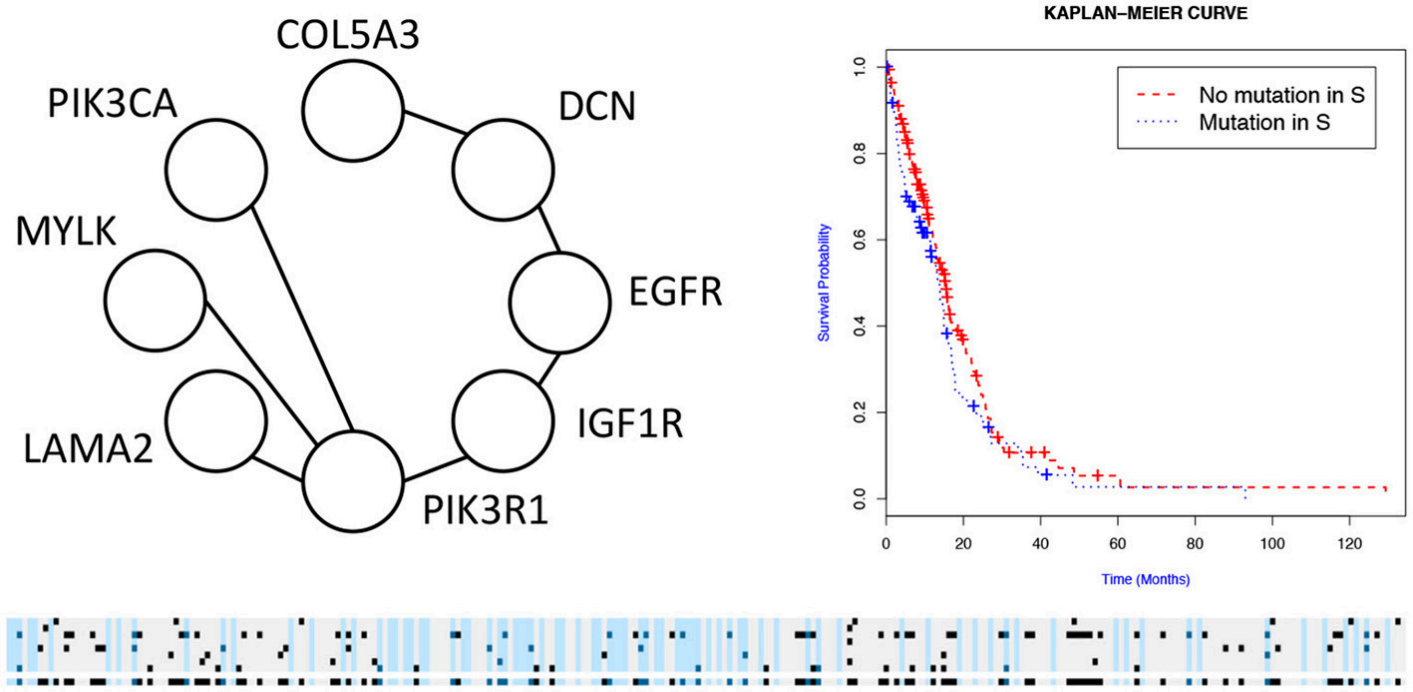
Subnetworks identified by NoMAS on GBM data. Subnetwork *S* associated with survival in GBM, Kaplan–Meier plot for the samples with mutations in *S* vs. samples with no mutation in *S*. The bottom panel shows the mutations in patients for the genes and the entire subnetwork (last row); patients with censored survival are in gray, other patients are in light blue; mutations in patients are show in dark color.

**Figure 5:**
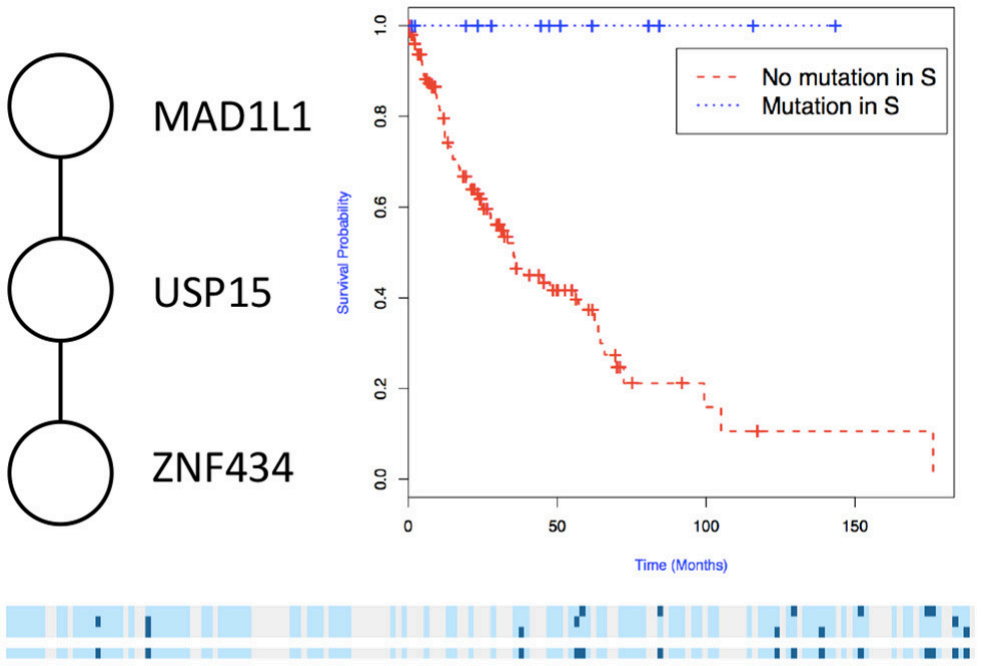
Subnetworks identified by NoMAS on LUSC data. Subnetwork *S* associated with survival in LUSC, Kaplan–Meier plot for the samples with mutations in *S* vs. samples with no mutation in *S*. The bottom panel shows the mutations in patients for the genes and the entire subnetwork (last row); patients with censored survival are in gray, other patients are in light blue; mutations in patients are show in dark color.

**Figure 6:**
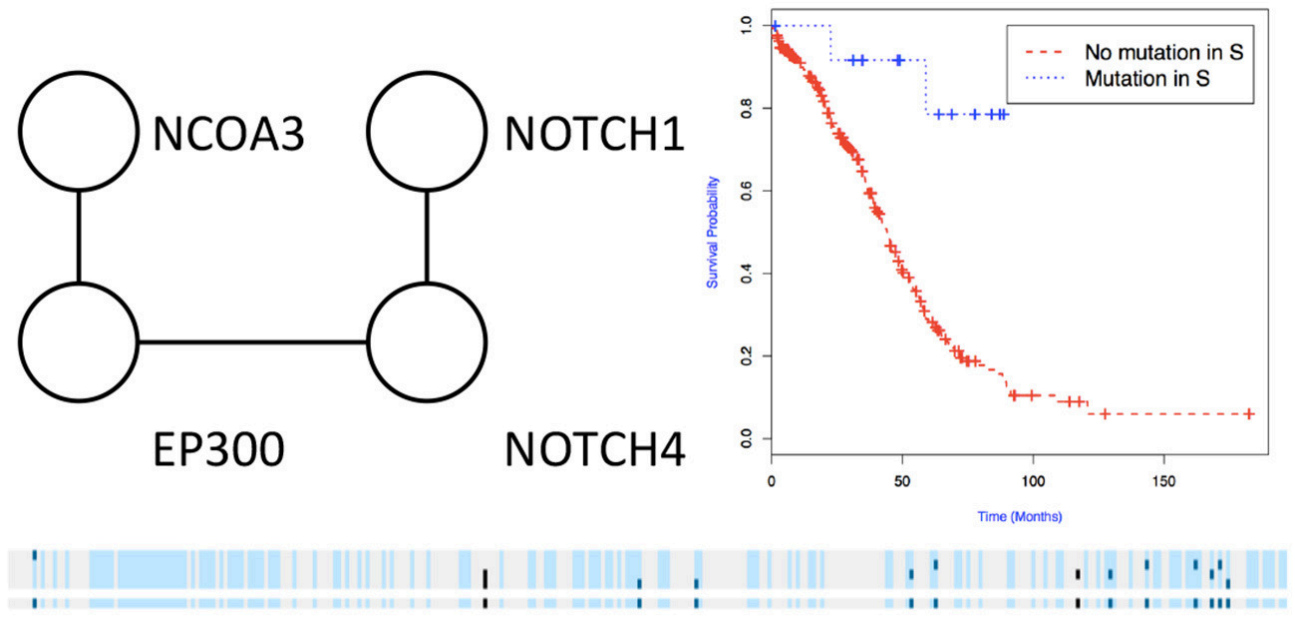
Subnetworks identified by NoMAS on OV data. Subnetwork *S* associated with survival in OV, Kaplan-Meier plot for the samples with mutations in *S* vs. samples with no mutation in *S*. The bottom panel shows the mutations in patients for the genes and the entire subnetwork (last row); patients with censored survival are in gray, other patients are in light blue; mutations in patients are show in dark color.

We then used the holdout approach to identify significant subnetworks for GBM, LUSC, and OV, considering the top-10 highest scoring subnetworks found in the training set and compute their *p*-value in the holdout set. We test all datasets using *k* = 3, 4, 8, 256 iterations of the color coding algorithm. As before, as pre-processing, genes mutated in < 3 samples were eliminated. NoMAS identified several subnetworks with significant association to survival. In GBM, for *k* = 8, NoMAS found the subnetwork including COL5A3, DCN, EGFR, IGF1R, LAMA2, MYLK, PIK3R1, and PIK3CA (*p* ≤ 0.05; Figure 4). None of the genes is associated with survival when considered in isolation. DCN, EGFR, IGF1R, PIK3R1 recur in various metabolic functions related to lipids and enzymes signaling and reception. These genes, together with PIK3CA, MYLK, and LAMA2, are involved in formation and maintenance of biological tissues, in cell movement and migration and cell protection organization. Moreover, EGFR, PIK3R1, and PIK3CA are well-known cancer genes. EGFR, IGF1R, LAMA2, MYLK, PIK3CA, PIK3R1, and MYLK are members of the focal adhesion pathway, whose dynamics are highly altered in cancer cells. In LUSC, NoMAS found the subnetwork including MAD1L1, USP15, and ZNF434 (*p* ≤ 0.03; Figure 5). None of the genes is associated with survival when considered in isolation. USP15 stabilizes MDM2, a well-known cancer gene, to regulate cancer-cell survival and mediates antitumor T cell responses (Zou et al., 2014), while increased expression of MAD1L1 is associated with poor prognosis in breast cancer (Sun et al., 2013). In OV, NoMAS identified the subnetwork including EP300, NCOA3, NOTCH1, and NOTCH4 (*p* ≤ 0.1; Figure 6). None of the genes is associated with survival when considered in isolation. These genes are part of a pathway related to RNA metabolic processes and have a role in regulation of epidermis development and cell differentiation within its layers. All genes are also linked to the thyroid hormone signaling pathway, that is related to cell death and DNA damage in ovarian cancer (Shinderman-Maman et al., 2017).

## 4. Discussion

In this work, we study the problem of identifying subnetworks of a large gene-gene interaction network that are associated with survival using mutations from large cancer genomic studies. Few methods have been proposed to identify groups of genes with mutations associated with survival in genomic studies. The work of Vandin et al. (2012a) combines mutations and survival data with interaction information using a diffusion process on graphs starting from gene scores derived from *p*-values of individual genes, but did not consider the problem of directly identifying groups of genes associated with survival. The work of Reimand and Bader (2013) combines mutation information and patient survival to identify subnetworks of a kinase-substrate interaction network associated with survival. It only focuses on phosphorylation-associated mutations, and the approach is based on a local search algorithm that builds a subnetwork by starting from one seed vertex and then greedily adding neighbors (at distance at most 2) from the seed, extending the approach used in different types of network analyses (Chuang et al., 2007). A similar greedy approach is used by Wu and Stein (2012) to identify groups of genes significantly associated with survival in cancer from gene expression data. For gene expression studies, Chowdhury et al. (2011) proposes an approach to enumerate dysregulated subnetworks in cancer based on an efficient search space pruning strategy, inspired by previous work on the identification of association rules in databases (Smyth and Goodman, 1992). Patel et al. (2013) uses the general approach described in Chowdhury et al. (2011) to identify subnetworks of genes with expression status associated to survival.

Color-coding is a probabilistic method that was originally described for finding simple paths, cycles and other small subnetworks of size *k* within a given network (Alon et al., 1994). The core of the color-coding technique is the assignment of random colors to the vertices, as a result of which the search space can be reduced, by restricting the subnetworks under consideration to *colorful* ones, those in which each vertex has a distinct color. For the identification of colorful subnetworks, dynamic programming is employed. The process is repeated until the desired subnetwork has been identified, that is having been colorful at least once, with high probability. When the dynamic programming algorithm is polynomial in *n* and the subnetworks being screened are of size *k*∈*O*(log*n*), the overall running time of the color-coding method too remains polynomial in *n*. Color-coding has been previously used to count or search for subgraphs of large interaction networks (Alon et al., 2008; Bruckner et al., 2010). Color-coding has also been used to identify groups of interacting genes in an interaction network that are associated with a phenotype of interest, but restricted to additive scores for sets of genes (i.e., the score of a set is the sum of the scores of the single genes); for example, Dao et al. (2011) uses color-coding to find optimally discriminative subnetwork markers that predict response to chemotherapy from a large interaction network by defining a single gene score as −log_10_*d*(*g*), where *d*(*g*) is the discriminative score for gene *g* (i.e., a measure of the ability of *g* to discriminate two classes of patients); similarly, Hormozdiari et al. (2015) uses color-coding to find groups of interacting genes with discriminative mutations in case-control studies, using as gene score the −log_10_ of the *p*-value from the binomial test of recurrence of mutations in the cases (while limiting the number of mutations in the controls).

In this work we formally define the associated computational problem, that we call the max connected *k*-set log-rank problem, by using as score for a subnetwork the test statistic of the log-rank test, one of the most widely used statistical tests to assess the significance in the difference in survival among two populations. We prove that the max connected *k*-set log-rank problem is NP-hard in general, and is NP-hard even when restricted to graphs with at least one node of large degree. We develop a new algorithm, NoMAS, based on the color-coding technique, to efficiently identify high-scoring subnetworks associated with survival. We prove that even if our algorithm is not guaranteed to identify the optimal solution with the probability given by the color-coding technique (due the non-additivity of our scoring function), it does identify the optimal solution with the same guarantees given by the color-coding technique when the data comes from a reasonable model for mutations and independently of the survival data. Using simulated data, we show that NoMAS is more efficient than the exhaustive algorithm while still identifying the optimal solution, and that our algorithm will identify subnetworks associated with survival when sample sizes larger than most currently available ones, but still reasonable, are available.

We use cancer data from three cancer studies from TCGA to compare NoMAS to approaches based on single gene scores and to greedy methods similar to ones proposed in the literature for the identification of subnetworks associated with survival and for other problems on graphs. Our results show that NoMAS identifies subnetworks with stronger association to survival compared to other approaches, and allows the correct estimation of *p*-values using a permutation test. Moreover, in two datasets NoMAS identifies two subnetworks associated with survival containing genes previously reported to be important for prognosis in the same cancer type as well as novel genes, while no gene is significantly associated with survival when considered in isolation.

There are many directions in which this work can be extended. First, we only considered single nucleotide variants and indels in our analysis; we plan to extend our method to consider more complex variants (e.g., copy number aberrations and differential methylation) in the analysis. Second, we believe that our algorithm and its analysis could be extended to the identification of subnetworks associated with clinical parameters other than survival time and to case-control studies, but substantial modifications to the algorithm and to its analysis will be required. Third, this work considers the log-rank statistic as a measure of association with survival; another popular test in survival analysis is the use of Cox's regression model (Kalbfleisch and Prentice, 2002). The two tests are identical in the case of two populations, therefore our algorithm identifies subnetworks with high score w.r.t. Cox's regression model as well. However, Cox's regression model allows for the correction for covariates (e.g., gender, age, etc.) in the analysis of survival data. A similar approach could be obtained by stratifying the patients in the log-rank test, but how to efficiently identify subnetworks, and in general combinations of genomic features, associated with survival while correcting for covariates remains a challenging open problem. Fourth, genomic regions other than genes (e.g., regulatory regions) or even other regulatory elements (e.g., microRNAs regulating the expression of driver genes) may be important for survival: the incorporation in our method of alterations in such regions and elements is an interesting direction for future research. Finally, in some studies the information regarding tumor (sub)clones and their mutations may be available: how to properly integrate such information in our analyses is a challenging direction for further investigation.

## Author Contributions

FV conceived and designed the study. FA, TH, and FV designed the algorithms, performed the analyses, and wrote the manuscript. FA and TH wrote the software.

### Conflict of Interest Statement

The authors declare that the research was conducted in the absence of any commercial or financial relationships that could be construed as a potential conflict of interest.

## Footnotes

^1^In the literature two different standard deviations (corresponding to two related but different null distributions, permutational and conditional) have been proposed for the normal approximation of the distribution of the log-rank statistic; we have previously shown (Vandin et al., 2015) that the one we use here (corresponding to the permutational distribution) is more appropriate for genomic studies.

^2^The implementation of NoMAS is available at https://github.com/VandinLab/NoMAS

^3^http://compbio-research.cs.brown.edu/pancancer/hotnet2/
