# Supplementary material for: NoMAS: A Computational Approach to Find Mutated Subnetworks Associated With Survival in Genome-Wide Cancer Studies
Source: Front Genet. 2019 Apr 10;10:265. doi: 10.3389/fgene.2019.00265 (PMC6468148; doi:10.3389/fgene.2019.00265)
Supplement: Supplementary file 1 [file Data_Sheet_1.PDF]

NoMAS: A Computational Approach to Find Mutated Subnetworks Associated with Survival in Genome-Wide Cancer Studies

Federico Altieri* Tommy Hansen† Fabio Vandin *,‡
vandinfa@dei.unipd.it

Supplemental Material

A Proof Sketches

Proposition 1. *For $i = 1, 2, \ldots, m$, let $w_i = c_i - \sum_{j=1}^{i} \frac{c_j}{m-j+1}$. Then*

$$\sum_{j=1}^{m} c_j \left(x_j - \frac{m_1 - \sum_{i=1}^{j-1} x_i}{m-j+1} \right) = \sum_{j=1}^{m} w_j x_j.$$

Proof (Sketch).

$$\begin{aligned}
\sum_{j=1}^{m} w_j x_j &= \sum_{j=1}^{m} \left(c_j - \sum_{i=1}^{j} \frac{c_i}{m-i+1} \right) x_j \\
&= \left(\sum_{j=1}^{m} c_j x_j \right) - \left(\sum_{j=1}^{m} x_j \sum_{i=1}^{j} \frac{c_i}{m-i+1} \right) \\
&= \left(\sum_{j=1}^{m} c_j x_j \right) - \left(\sum_{j:c_j \neq 0} \frac{\sum_{i=j}^{m} x_i}{m-j+1} \right) \\
&= \left(\sum_{j=1}^{m} c_j x_j \right) - \left(\sum_{j=1}^{m} c_i \frac{m_1 - \sum_{i=1}^{j-1} x_i}{m-j+1} \right) \\
&= \sum_{j=1}^{m} c_j \left(x_j - \frac{m_1 - \sum_{i=1}^{j-1} x_i}{m-j+1} \right) = V(\mathbf{x}).
\end{aligned}$$

□

Theorem 1. *The max k-set log-rank problem is NP-hard.*

Proof (Sketch). The reduction is from the minimum set cover problem. In particular, we will show that if we can find a set $\mathcal{S}$ with $|\mathcal{S}|$ maximizing $w'(\mathcal{S})$ in polynomial time, then we can test (in polynomial time) if there is a set cover of cardinality k. This implies that one could find the size of the minimum set cover in polynomial time, that is an NP-hard problem.

*Department of Information Engineering, University of Padova, Padova (Italy).
†Department of Mathematics and Computer Science, University of Southern Denmark, Odense (Denmark).
‡Corresponding author.

In the minimum set cover problem, one is given elements $e_1, \dots, e_n$, where each element $e_i, 1 \leq i \leq n$ is a subset of a universe set $\mathcal{U}$, with $|\mathcal{U}| = m$. The goal is to find the minimum cardinality subset $\mathcal{C} \subset \{e_1, \dots, e_n\}$ such that $\cup_{e \in \mathcal{C}} = \mathcal{U}$.

Given an instance of the minimum set cover problem, we build an instance of the max k-set log-rank as follows. For each element $e_i, 1 \leq i \leq n$, we have a gene g_i, with $\mathcal{G} = \{g_1, \dots, g_n\}$. The set $\mathcal{P}$ of patients has cardinality $4|\mathcal{U}|$. $\mathcal{P}$ is partitioned into two sets $\mathcal{P}_1$ and $\mathcal{P}_2$, with $\mathcal{P}_1 \cap \mathcal{P}_2 = \emptyset$ and $\mathcal{P} = \mathcal{P}_1 \cup \mathcal{P}_2$. Moreover we have $|\mathcal{P}_1| = \mathcal{U}$ and $|\mathcal{P}_2| = 3\mathcal{U}$, and the survival time of all patients in $\mathcal{P}_1$ is lower than the survival time of all patients in $\mathcal{P}_2$. In addition, no patient of $\mathcal{P}_1$ is censored, while all patients in $\mathcal{P}_2$ are censored. The patients of $\mathcal{P}_1$ correspond to the elements of $\mathcal{U}$, and gene g_i is mutated in patients $e_i \subset \mathcal{U}$.

We now show that there is a minimum set cover of cardinality k if and only if $\max_{\mathcal{S} \subset \mathcal{G}, |\mathcal{S}| = k} w'(\mathcal{S}) = \frac{\frac{m}{4} - \sum_{j=1}^{m/4} \frac{1}{m-j+1}}{\sqrt{9m^2/16}}$. In particular, we will show that the maximum log-rank statistic is obtained when $x_i = 1$ for all $1 \leq i \leq \frac{m}{4}$ and $x_i = 0$ for all $\frac{m}{4} < i \leq n$, that can be achieved if and only if there is a set cover of cardinality k. (Note that m is divisible by 4 by construction.)

To prove the above, it is enough to show the following:

i) $w_i > 0$ for $1 \leq i \leq \frac{m}{4}$;

ii) for a fixed $m_1 \leq \frac{m}{4}$, the maximum weight is given by $\frac{\sum_{i=1}^{m_1} w_i}{\sqrt{m_1(m-m_1)}}$;

iii) for all $1 \leq j \leq \frac{m}{4} - 1$: $\frac{\sum_{i=1}^{j} w_i}{\sqrt{j(m-j)}} \leq \frac{\sum_{i=1}^{j+1} w_i}{\sqrt{(j+1)(m-(j+1))}}$.

We first note that $w_i > w_{i+1}$ for $1 \leq i < \frac{m}{4}$.

$$w_i - w_{i+1} = 1 - \sum_{j=1}^{i} \frac{1}{m-j+1} - 1 + \sum_{j=1}^{i+1} \frac{1}{n-j+1} = \frac{1}{m-i} > 0.$$

To prove i) above it is then enough to prove that $w_{\frac{m}{4}} > 0$.

$$w_{\frac{m}{4}} = 1 - \sum_{j=1}^{m/4} \frac{1}{m-j+1} = 1 - \sum_{j=\frac{3m}{4}+1}^{m} \frac{1}{j} = 1 - H(m) + H\left(\frac{3m}{4}\right).$$

where $H(m)$ is the m-th harmonic number. Since $H(m) \leq \ln m + \gamma + \frac{1}{2m}$, with $\gamma \leq 0.58$ constant, for m large enough, and $H(m) \geq \ln m$, we have:

$$w_{\frac{m}{4}} \geq 1 - \ln m - \gamma - \frac{1}{2m} + \ln \frac{3m}{4} \geq 1 - \ln \frac{4}{3} - \gamma - \frac{1}{2m} > 0.1 - \frac{1}{m} > 0 \quad (1)$$

for m large enough.

ii) follows immediately from i) and from $w_i > w_{i+1}$ for $1 \leq i < \frac{m}{4}$ (since fixed m_1, the denominator $\sqrt{m_1(m-m_1)}$ is fixed).

We now prove iii). We first derive an alternative way to write $\sum_{i=1}^{j} w_i$:

$$\begin{aligned}
\sum_{i=1}^{j} w_i &= \sum_{i=1}^{j}\left(1-\sum_{\ell=1}^{i}\frac{1}{m-\ell+1}\right)\\
&= j-\sum_{i=1}^{j}\sum_{\ell=1}^{i}\frac{1}{m-\ell+1}\\
&= j-\sum_{\ell=1}^{j}\frac{j-\ell-1}{m-\ell+1}\\
&= j-\sum_{\ell=0}^{j-1}\frac{j-\ell}{m-\ell}\\
&= j-\sum_{\ell=0}^{j-1}\frac{j}{m-\ell}+\sum_{\ell=0}^{j-1}\frac{\ell}{m-\ell}\\
&= j\left(1-\sum_{\ell=0}^{j-1}\frac{1}{m-\ell}\right)+\sum_{\ell=1}^{j-1}\frac{\ell}{m-\ell}.
\end{aligned}$$

Analogously

$$\sum_{i=1}^{j+1} w_i = (j+1)\left(1-\sum_{\ell=0}^{j}\frac{1}{m-\ell}\right)+\sum_{\ell=1}^{j}\frac{\ell}{m-\ell}$$

We now prove that

$$\frac{\sum_{\ell=1}^{j}\frac{\ell}{m-\ell}}{\sqrt{(j+1)(m-(j+1))}} \geq \frac{\sum_{\ell=1}^{j-1}\frac{\ell}{m-\ell}}{\sqrt{j(m-j)}}$$

and that

$$\frac{(j+1)\left(1-\sum_{\ell=0}^{j}\frac{1}{m-\ell}\right)}{\sqrt{(j+1)(m-(j+1))}} \geq \frac{j\left(1-\sum_{\ell=0}^{j-1}\frac{1}{m-\ell}\right)}{\sqrt{j(m-j)}}$$

that together imply iii).

We start from the first inequality. The proof is by induction on j. The base case $j = 1$ is proved by substitution: the left hand side is > 0 while the right hand side in 0. Now let us assume that the inequality is true for all values up to $j - 1 \geq 1$: we prove that the inequality is correct for j.

$$\begin{aligned}
\frac{\sum_{\ell=1}^{j}\frac{\ell}{m-\ell}}{\sqrt{(j+1)(m-(j+1))}} &= \frac{\sum_{\ell=1}^{j-1}\frac{\ell}{m-\ell}+\frac{j}{m-j}}{\sqrt{(j+1)(m-(j+1))}}\\
&\geq \frac{\frac{\sqrt{j(m-j)}}{\sqrt{(j-1)(m-(j-1))}}\sum_{\ell=1}^{j-2}\frac{\ell}{m-\ell}+\frac{j}{m-j}}{\sqrt{(j+1)(m-(j+1))}}
\end{aligned}$$

where the second inequality follows from the inductive hypothesis. Now note that

$$\frac{\sqrt{j(m-j)}}{\sqrt{(j-1)(m-(j-1))}}\sum_{\ell=1}^{j-2}\frac{\ell}{m-\ell} \geq \frac{\sqrt{(j+1)(m-(j+1))}}{\sqrt{j(m-j)}}\sum_{\ell=1}^{j-2}\frac{\ell}{m-\ell}$$

since the function

$$\frac{\sqrt{(j+1)(m-(j+1))}}{\sqrt{j(m-j)}}$$

is non-increasing in j for $j \leq \frac{m}{4}$. To complete the proof we need to show that $\frac{j}{m-j} \geq \frac{\sqrt{(j+1)(m-(j+1))}}{\sqrt{j(m-j)}} \frac{j-1}{m-j+1}$:

$$\begin{aligned}
\frac{j}{m-j} &\geq \frac{\sqrt{(j+1)(m-(j+1))}}{\sqrt{j(m-j)}} \frac{j-1}{m-j+1} \\
\frac{j(m-j+1)}{(j-1)(m-j)} &\geq \sqrt{\frac{(j+1)(m-j-1)}{j(m-j)}} \\
\frac{j^2(m-j+1)^2}{(j-1)^2(m-j)^2} &\geq \frac{(j+1)(m-j-1)}{j(m-j)} \\
j^3(m-j+1)^2 &\geq (j+1)(m-j-1)(j-1)^2(m-j).
\end{aligned}$$

Since $(m-j+1)^2 \geq (m-j-1)(m-j)$, we just need to prove $j^3 \geq (j+1)(j-1)^2$:

$$\begin{aligned}
j^3 &\geq (j+1)(j-1)^2 = j^3 - j^2 - j + 1 \\
j^2 + j &\geq 1
\end{aligned}$$

that is true for all $j \geq 1$.

The proof of

$$\frac{(j+1)\left(1 - \sum_{\ell=0}^{j} \frac{1}{m-\ell}\right)}{\sqrt{(j+1)(m-(j+1))}} \geq \frac{j\left(1 - \sum_{\ell=0}^{j-1} \frac{1}{m-\ell}\right)}{\sqrt{j(m-j)}}$$

is analogous. □

Theorem 2. *The max connected k-set log-rank problem on graphs with at least one node of degree $O\left(n^{\frac{1}{c}}\right)$, where $c > 1$ is constant, is NP-hard.*

Proof (Sketch). Take an instance of set cover with n elements. We can "encode" it in the neighbours of a node of degree n in a graph with n^c vertices, where $c > 0$ is a constant, using the same scheme used for Theorem 1. All other vertices have no mutations. Note that the reduction is polynomial. □

Proposition 2. *For every $k \geq 3$ there is a family of instances of the max connected k-set log-rank problem and colorings for which OPT is not found by our algorithm even if it is colorful.*

Proof (Sketch). Let the number of samples be $n = 8(k-1)$. The censoring information $\mathbf{c}$ is such that $c_i = 1$ for $1 \leq i \leq \frac{n}{4}$ and $c_j = 0$ for $\frac{n}{4}+1 \leq j \leq n$. From Theorem 1 we get that all weights $w_i > 0$ for $1 \leq i \leq \frac{n}{4}$. Let $\mathcal{I}$ be a tree with one internal vertex v_0 and $k+1$ leaf vertices $\{v_1, v_2, \ldots, v_{k-1}, \bar{v_1}, \bar{v_2}\}$. Consider a coloring $\mathcal{C}$ in which $\mathcal{C}(v_i)$ are distinct for $0 \leq i \leq k-1$ and $\mathcal{C}(v_j) = \mathcal{C}(\bar{v_j})$ for $1 \leq j \leq 2$. Let $\sigma(v)$ be the set of weights for vertex v, i.e containing a weight for each sample mutated in the gene associated with v. Assign the weights such that $\sigma(v_0) = \emptyset$, $\sigma(v_i) = \{w_i, w_{k-1+i}\}$ and $\sigma(\bar{v}_i) = \{w_1, w_2\}$. Note that for any $k \geq 3$ the optimal connected subnetwork $OPT = \mathcal{S} = \{v_0, v_1, \ldots, v_{k-1}\}$ since $\sigma(\mathcal{S}) = \{w_1, w_2, \ldots, w_{n/4}\}$. By construction OPT is colorful.

The idea of the construction is to have two *bad* colors. A color c is bad if it is assigned to two vertices. The vertex in OPT with color c is a *good* vertex, while the vertex with color c not in OPT is a *bad*

vertex. In our construction v_1 and v_2 are good vertices and $\bar{v_1}$ and $\bar{v_2}$ are bad vertices. Recall that our algorithm combines two subnetworks that are connected by an edge, thus every subnetwork of size ℓ must be a combination of a leaf v_i and some subnetwork $W(T, v_0)$ of size $\ell - 1$. To generate OPT, at some point we will have that v_i is one of the good vertices while $W(T, v_0)$ contains the other good vertex. We will show that this cannot happen. In particular we argue that $W(T, v_0)$ cannot contain only one bad color and be a subset of OPT. Without loss of generality, assume v_1 is the vertex with a bad color in $W(T, v_0)$. Consider the time it is added to $W(T, v_0)$ by combination of some $W(Q, v_0) \setminus \{v_1, v_2\}$ and $W(\{\mathcal{C}(v_1)\}, v_1)$. However, our algorithm will choose to combine with $\bar{v_1}$ in stead of v_1 because $\bar{v_1}$ yields the largest increase in the normalized log-rank statistic. To see this, note that v_1 and $\bar{v_1}$ both add two weights to $\sigma(W(Q, v_0))$ that are not already in $\sigma(W(Q, v_0))$. Both options therefore have the same number of mutations, and their normalized log-rank statistic can be compared by simply comparing their log-rank statistic. By construction $\sigma(\bar{v_0})$ contains the two largest weights, hence it yields the larger log-rank statistic.

□

Theorem 3. *For any optimal colorful connected subnetwork $\mathcal{S}$ of size $k \geq 3$ and any algorithm $\mathcal{A}$ which obtains subnetworks with colorsets of cardinality i by combining 2 subnetworks with colorsets of cardinality $< i$, by adding 3 neighbors to $\mathcal{S}$ we have that $\mathcal{A}$ may not discover S .*

sketch. Let the k vertices of OPT be deemed *good* vertices. For each of three of the vertices in OPT we add a *bad* copy, so that the good vertex v and the bad vertex $\bar{v}$ have the same color and the same connectivity to the vertices in $OPT \setminus \{v\}$. By definition of $\mathcal{A}$, $\mathcal{S}$ is found by combining two subnetworks of cardinality $< k$, and because there are three good vertices in OPT, one of these subnetworks of cardinality $< k$ will contain at least two good vertices. We show that an evil adversary can ensure that two subnetworks $\mathcal{S}_1$ and $\mathcal{S}_2$, both being entries in W and each containing a good vertex, will never be combined by $\mathcal{A}$.

The combination of $\mathcal{S}_1$ and $\mathcal{S}_2$ will happen across a specific edge in the graph between one vertex $v_1 \in \mathcal{S}_1$ and one vertex $v_2 \in \mathcal{S}_2$. If v_2 is a good vertex then there will be another subnetwork $\bar{\mathcal{S}_2}$ in W with the same colorset as $\mathcal{S}_2$, namely in the column corresponding to the bad vertex $\bar{v_2}$, and since the connectivities of v_2 and $\bar{v_2}$ to OPT are the same, $\mathcal{A}$ must select one of them. Due to the fact that $|\mathcal{S}_1 \cup \mathcal{S}_2| < k$ the adversary will be able to plant mutations so that $\bar{\mathcal{S}_2}$ is chosen over $\mathcal{S}_2$. If v_2 is neither a good nor a bad vertex the same argument can be made to show that the adversary can ensure that $\mathcal{S}_2$ will not contain any good vertices. □

The following is a result that we need to prove the performance of NoMAS under the Planted Subnetwork Model.

Proposition 3. *For every censoring vector c: $\sum_{i=1}^{m} w_i = 0$.*

Proof (Sketch). When $c_i = 1$ for all $1 \le i \le m$, the we have

$$\begin{aligned}
\sum_{i=1}^{m} w_i &= \sum_{i=1}^{m}\left(c_i - \sum_{j=1}^{i}\frac{c_j}{m-j+1}\right)\\
&= \sum_{i=1}^{m}\left(1 - \sum_{j=1}^{i}\frac{1}{m-j+1}\right)\\
&= m - \sum_{i=1}^{m}\sum_{j=1}^{i}\frac{1}{m-j+1}\\
&= m - \sum_{i=1}^{m} i\frac{1}{i}\\
&= m - m\\
&= 0.
\end{aligned}$$

When one c_i is switched to the value 1, we have that the weight changes by a factor:

$$-1 + \sum_{j=i}^{m}\frac{1}{m-i+1} = 0 \qquad (2)$$

where the -1 is subtracted to w_i, while the value $\frac{1}{m-i+1}$ is summed (i.e., not subtracted) to all terms w_j with $j \ge i$. Therefore, any change to the censoring vector leaves $\sum_{i=1}^{m} w_i = 0$. $\square$

Using the above, we can prove the following.

Theorem 4. *Let M be a mutation matrix corresponding to m samples from the Planted Subnetwork Model. If $m \in \Omega\left(k^4(k+\varepsilon)\ln n\right)$ for a given constant $\varepsilon > 0$ and $O\left(\ln(1/\delta)e^k\right)$ color-coding iterations are performed, then our algorithm identifies the optimal solution $\mathcal{D}$ to the max connected k-set log-rank with probability $\ge 1 - \frac{1}{n^{\varepsilon}} - \delta$.*

Proof (Sketch). Assume that $\mathcal{D}$ is colorful. We prove that if NoMAS has build a subnetwork (with $1 \le i < k$ vertices) consisting of vertices of $\mathcal{D}$ only, then if $m \in \Omega\left(k^2(k+\varepsilon)\ln n\right)$, NoMAS will expand such solution by only using vertices in $\mathcal{D}$. Since NoMAS starts to build solutions from each vertex in $\mathcal{D}$, this proves that NoMAS identifies the optimal solution. We show this by proving that *any* set $\mathcal{C} \subset \mathcal{G} \setminus \mathcal{D}$, when added to *any* subset $\mathcal{S} \subset \mathcal{D}$, does not provide an improvement in the score as just adding one of the genes in $\mathcal{D}$.

From the properties of the Planted Subnetwork Model (PSM), we have that if $\mathcal{S}$ is a subset of $\mathcal{D}$, then $w(S) \ge \frac{c'm}{k}$, where c' is a constant > 0. For a set $\mathcal{C} \subset \mathcal{G} \setminus \mathcal{D}$, we can consider it as a "metagene" that is mutated with a certain probability q (constant) in each sample, where q depends on the genes in $\mathcal{C}$.

From Property 3, we have that $\mathbf{E}[w(\mathcal{S} \cup \mathcal{C}) - w(\mathcal{S})] = -qw(\mathcal{S}) \le -q\frac{c'm}{k}$, since the sum of all weights w_i is 0 and $\mathcal{C}$ adds weights from a set of weights that must sum to $-w(\mathcal{S})$. From the properties of PSM, for a gene $g \in \mathcal{D} \setminus \mathcal{S}$ we have $w(\mathcal{S} \cup \{g\}) - w(\mathcal{S}) \ge \frac{c''m}{k}$, with $c'' > 0$ constant. Note that $w(\mathcal{S} \cup \mathcal{C}) - w(\mathcal{S})$ is the sum of independent random variables, and each random variable can change the value of $w(\mathcal{S}\cup\mathcal{C}) - w(\mathcal{S})$ by a value $< m$. Moreover, the number of samples in which $\mathcal{C}$ can have mutations while $\mathcal{S}$ does not is at least $\frac{m}{k}$ and at most m. We can therefore use Hoeffding inequality to bound the probability that $w(\mathcal{S} \cup \mathcal{C}) > w(\mathcal{S} \cup \{g\})$ as follows:

$$\begin{aligned}
\mathbf{Pr}(w(\mathcal{S} \cup \mathcal{C}) > w(\mathcal{S} \cup \{g\})) &= \mathbf{Pr}(w(\mathcal{S} \cup \mathcal{C}) - w(\mathcal{S}) > w(\mathcal{S} \cup \{g\}) - w(\mathcal{S}))\\
&\le e^{-d((\frac{m}{k})^2(\frac{m}{k})^2/m^3}\\
&\le \frac{1}{n^{k+\varepsilon}}
\end{aligned}$$

for an appropriate constant $d > 0$ and for $m \in \Omega\left(k^4(k+\varepsilon)\ln n\right)$. By union bound on all sets $\mathcal{C}$ of cardinality $\leq k$, we have that $\mathbf{Pr}(w(\mathcal{S} \cup \mathcal{C}) > w(\mathcal{S} \cup \{g\})) \leq \frac{1}{n^{k+\varepsilon}} n^k = \frac{1}{n^{\varepsilon}}$. Therefore, when $m \in \Omega\left(k^4(k+\varepsilon)\ln n\right)$ and $\mathcal{D}$ is colorful, then NoMAS finds $\mathcal{D}$ with probability $\geq 1 - \frac{1}{n^{\varepsilon}}$. The probability that $\mathcal{D}$ is not colorful in any of the $O\left(\ln(1/\delta)e^k\right)$ color-coding iterations is $\leq \delta$. Therefore, by union bound the probability that NoMAS does not identify $\mathcal{D}$ when $m \in \Omega\left(k^4(k+\varepsilon)\ln n\right)$ is $\leq \delta + \frac{1}{n^{\varepsilon}}$, and the result follows. □

B Modifications to NoMAS

We design two modifications of NoMAS that can solve some easy cases where NoMAS may not identify the highest scoring solution due to its subnetwork merging strategy:

i) we merge a subnetwork $W(T,u)$ not only with subnetworks $W(R,v)$ where v is a neighbor of u, but with subnetworks $W(R,w)$ where w is a neighbor of *any* vertex in $W(T,u)$;

ii) in $W(T,u)$, we store $\ell > 1$ different colorful subnetworks containing u and with colorset T, leading to $\leq \ell^2$ choices for combining two entries of W and a corresponding ℓ^2 increase in the time complexity of the algorithm.

We note that the time complexity required by modification i) above is still polynomial at most a factor $|V|^2/|E| \in \Omega(n)$ larger than that of NoMAS. We note that both modifications find the optimal solution in the problem instance of Proposition 2, while the second one will find the optimal solution in the problem instance of Theorem 3 if ℓ is large enough. The second modification was run using $\ell = 5$ in our experiments and storing in $W(T,u)$ the $\leq \ell$ highest scoring subnetworks in $\mathcal{S}'(T,u)$.

C Greedy algorithms

We considered three different greedy strategies for the max connected k-set log-rank problem. All three algorithms build solutions starting from each node $u \in G$ in iterations by adding nodes to the current solution $\mathcal{S}$, and differ in the way the enlarge the current subnetwork $\mathcal{S}$ of size $1 \leq i < k$. The first, `Greedy1`, screens all vertices at distance 1 to $\mathcal{S}$ and adds the one that results in the best subnetwork of size $i+1$. The second, `GreedyK`, considers all vertices at distance $\leq k-i$ to $\mathcal{S}$, and enforces connectivity by greedily constructing a path from the selected vertex to a vertex in $\mathcal{S}$. The third, `GreedyDFS`, traverses shortest paths from $\mathcal{S}$ to every vertex at distance $\leq k-i$ by a depth-first search. The vertices on some shortest path of length $j \leq k-i$ which improved $\mathcal{S}$ the most are added to obtain a subnetwork of size $i+j$.

D Pseudo code for NoMAS

The pseudo code for NoMAS is divided into three algorithms. First, algorithm 1 highlights the overall color-coding scheme. Second, algorithm 2 describes how the dynamic programming table W is computed in order of increasing colorset group sizes. Finally, algorithm 3 details the process of computing the subnetwork at a specific entry in W. It is assumed that the undirected graph $G(V, E)$, the mutation matrix M and the survival information $\mathbf{x}, \mathbf{c}$ are globally known. As a companion piece to algorithm 3, figure 1 visualizes the method used for combining two previously computed entries of W.

Algorithm 1: NoMAS(k, δ)

```
best ← nil
for i ← 1 to ln(1/δ)e^k do
    Color the vertices of G with k colors uniformly at random
    W ← FillTable(k)
    best ← argmax_{∀T∀v : W(T,v)∈W} {w(W(T,v))}
return best
```

Algorithm 2: FillTable(k)

```
W ← empty table with dimensions (2^k − 1) × |V|
for each vertex u ∈ V do
    for each color α among the k colors do
        if the color of u is α then
            W({α}, u) ← {u}
        else
            W({α}, u) ← nil
for i ← 2 to k do
    /* The following may be distributed among N ≤ |V| processors */
    for each vertex u ∈ V do
        for each colorset T of size i do
            W(T, u) ← ComputeEntry(T, u)
return W
```

Algorithm 3: ComputeEntry(T, u)

```
best ← nil
for each neighbor v of u do
    for each colorset Q s.t. Q ⊂ T and Q ≠ ∅ do
        R ← T \ Q
        candidate ← W(Q, u) ∪ W(R, v)
        best ← arg max{w(candidate), w(best)}
return best
```

Modifications The two proposed modifications to NoMAS differ from NoMAS in their method for computing an entry of W. Algorithm 4 describes modification i, while algorithm 5 details modification ii. Both algorithms should be seen as replacements for algorithm 3 of the unmodified version of NoMAS. Figure 5 visualizes the combination strategy of algorithm 4 (note the difference from figure 1).

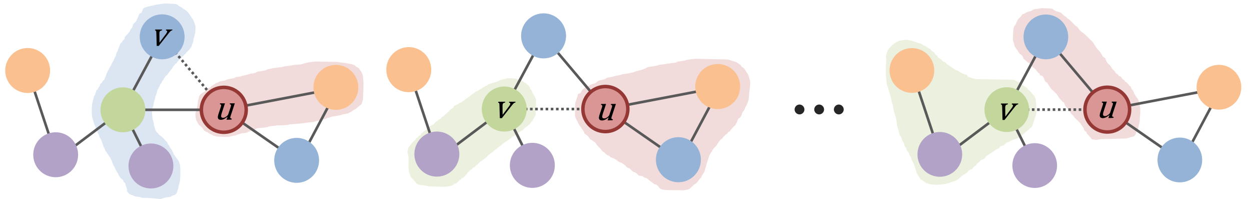

Figure 1: Examples of several pairs of colorful connected subnetworks $W(Q,u)$ and $W(R,v)$ considered by NoMAS when computing the entry $W(T,u)$ for a colorset T of size 5. In each example a subnetwork containing u are combined with a subnetwork containing a neighbor v of u, in order to obtain a subnetwork with colorset $T = Q \cup R$ such that $Q \cap R = \emptyset$. The dotted edge is the one connecting the two subnetworks (the edge is always connected to u).

Algorithm 4: MODIFICATIONI(T, u)

---

best $\leftarrow$ **nil**
for each colorset Q s.t. $Q \subset T$ and $Q \neq \emptyset$ **do**
  for each neighbor w of a vertex in $W(Q,u)$ **do**
    $R \leftarrow T \setminus Q$
    candidate $\leftarrow W(Q,u) \cup W(R,w)$
    best $\leftarrow \arg\max\{w(\text{candidate}), w(\text{best})\}$
return best

Algorithm 5: MODIFICATIONII(T, u)

---

candidates $\leftarrow \emptyset$
for each neighbor v of u **do**
  for each colorset Q s.t. $Q \subset T$ and $Q \neq \emptyset$ **do**
    $R \leftarrow T \setminus Q$
    for each subnetwork $A \in W(Q,v)$ **do**
      for each subnetwork $B \in W(R,v)$ **do**
        candidates $\leftarrow$ candidates $\cup\{A \cup B\}$
best $\leftarrow$ the ℓ distinct highest scoring subnetworks in candidates
return best

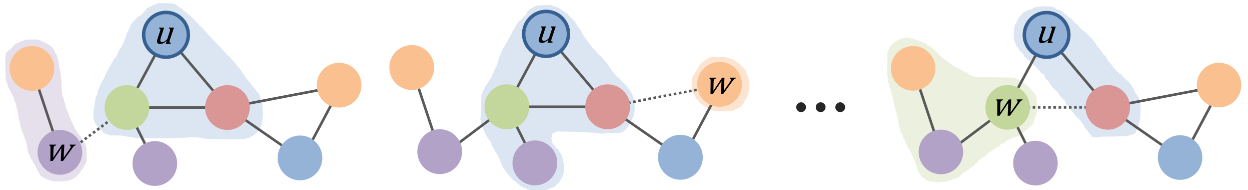

Figure 2: Examples of several pairs of colorful connected subnetworks $W(Q,u)$ and $W(R,w)$ considered by NoMAS with modification i when computing the entry $W(T,u)$ for a colorset T of size 5. In each example a subnetwork $W(Q,u)$ containing u are combined with a subnetwork containing a neighbor w of some vertex in $W(Q,u)$, in order to obtain a subnetwork with colorset $T = Q \cup R$ such that $Q \cap R = \emptyset$. The dotted edge is the one connecting the two subnetworks.

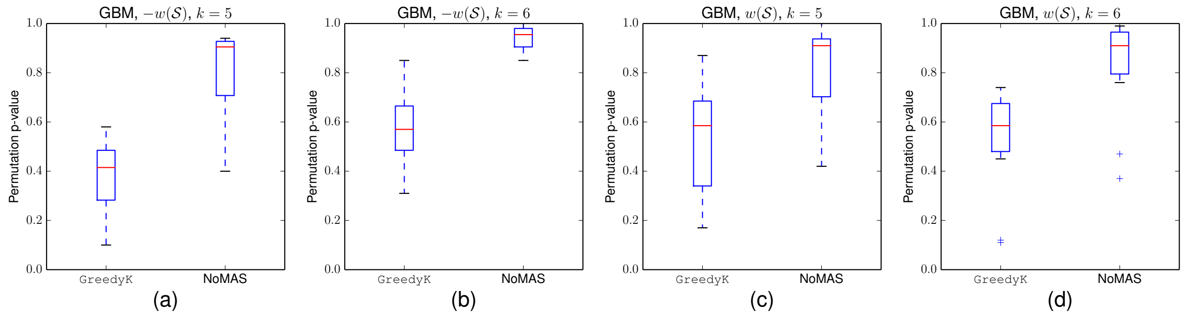

Supplementary Figure 1: p-values of the permutation test on the top 10 solutions identified by `GreedyK` for different values of k in both tail tests. The top 10 solutions on the permuted data are obtained using both `GreedyK` and NoMAS (with 32 color-coding iterations).

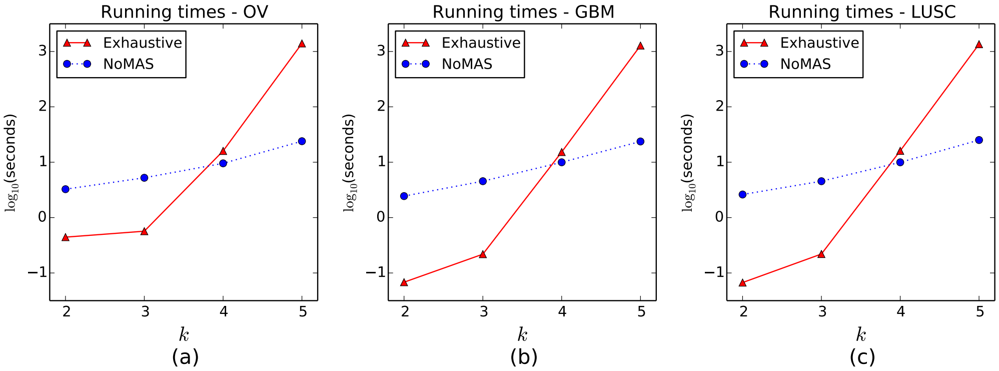

Supplementary Figure 2: Running time comparison between NoMAS and the exhaustive enumeration algorithm on three different cancer datasets. The running times of both algorithms are obtained using 40 processors. The running times for NoMAS account for 256 color-coding iterations and excludes the statistical assessment of the identified solutions.

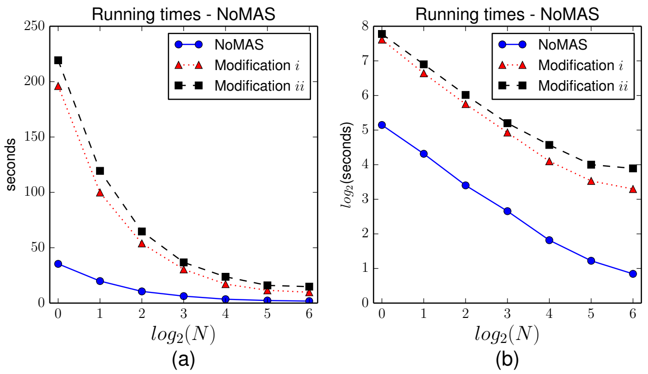

Supplementary Figure 3: Running times of NoMAS and the two modifications considered for varying numbers of processors N. The running times are for a single iteration for $k = 8$ and are obtained on the OV cancer data (a) The running times in seconds. (b) The running times in seconds on a logarithmic scale.

E Charts

All of the results displayed in the following charts are obtained using SNoMAS with error probability 0.1 and $k' = 4$. The seeds for SNoMAS are generated by using NoMAS with error probability 0.05 to find solutions for $k = 5$. Genes that are not mutated in any patients are removed from the gene-gene interaction network.

The p-values are approximated using permutation sampling with 10^8 samples. The permutation p-value ("Perm. p-value" on the charts) are obtained by permuting the data set 10 times and comparing the solutions identified on the permuted data with the ones from the real data. Permutation is performed by shuffling the identities of each gene, so that the network itself remains the same, but the symbols of the vertices change. A permutation p-value of 0.1 means that either a better solution was found in a one of the permuted datasets, or in *none* of the permuted dataset.

Charts are displayed for the GBM, OV and LAML datasets, in which the mutation matrices that contain both single nucleotide mutations (from TCGA) and copy number mutations. I have included both "old" and "new" TCGA mutations for GBM and OV. "Old" is the mutation matrices we used in this paper. "New" is the ones I generated recently. It is displayed on the second line in the top left corner of the charts. The TCGA mutation matrix for LAML is the one you sent me recently, which we called LAML_new. I have also added charts for the three data sets for the mutation matrices that contain only the copy number mutations. The mutations matrices are described in the captions.

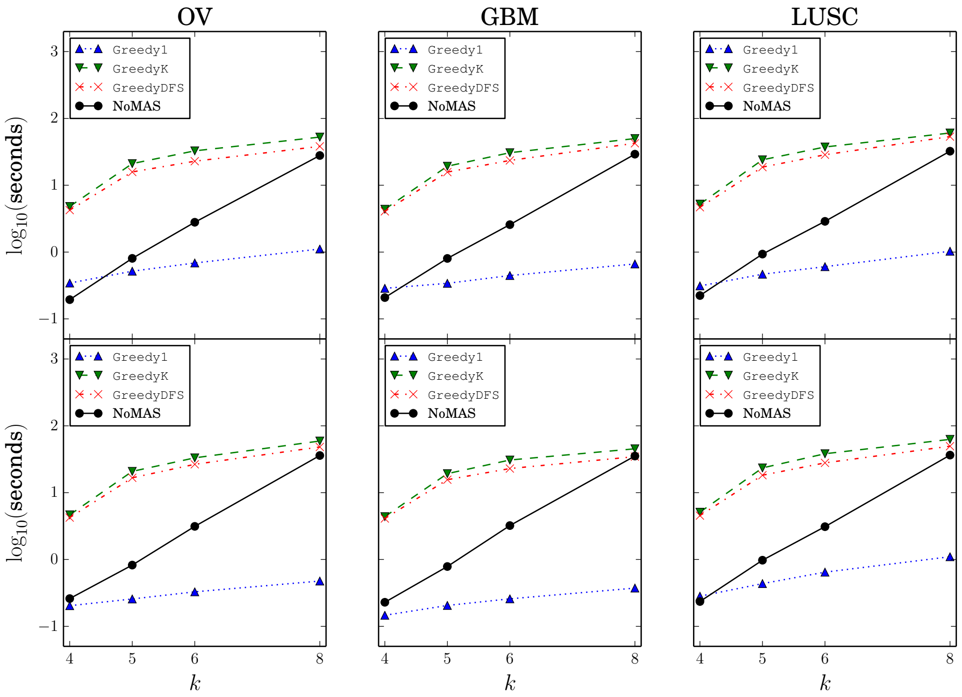

Supplementary Figure 4: Running times of the three greedy algorithms and a single color-coding iteration of NoMAS for varying values of k and on three different cancer data. Each of the algorithms are run on a single processor. The top panels show the times measured when maximizing the score $w(\mathcal{S})$, while the bottom panels show the times for maximizing the score $-w(\mathcal{S})$.
